# Case of Abnormal Position of the Intesties, &c., Translated from the German of Wolshofer

**Published:** 1848

**Authors:** 

**Affiliations:** Heubach


ARTICLE VI.

*Case of Abnormal Position of the Intestines in the opposite side of the Body.* By DR. WOLSHOFER, of Heubach. Translated from the German by DANIEL STAHL, M.D., of Quincy, Ill.

PROF. HERRICK — DEAR SIR:

The last No. of the "Illinois and Indiana Medical and Surgical Journal" contains an account of "*The case of Mr. Whitman,*" showing a congenital transposition of the organs of the thorax and abdomen, those in the normal condition situated in the left side, were in the right, and *vice versa*. This case is certainly highly interesting, the more so as the individual thus organized lived to the age of manhood, and might probably have lived to old age, if life had not been cut short by pleurisa. I saw, yesterday, a schoolmate of the late Mr. Whitman, who told me that he well remembered the "peculiar cough" spoken of in the Journal, even during his minority. This case, although rare, is not unique; and an accumulation of similar cases might probably give some future pathological philosopher data from which to deduce a principle, or to be in some other way, if not useful, at least interesting. Knowing the lively interest you take in any thing rare or useful in our profession, I need not apologise for troubling you with the subjoined translation from the German of a somewhat similar case to that of Mr. Whitman, related in the "Wurtemburger Correspondenz. Blatt, No. 13, 1842:

About the middle of September, 1841, the writer was called to a child of one year of age to give his professional advice concerning its condition. He found it to be a very feeble boy, of livid color, and learned, on inquiry, that the child had this bluish color of the skin from his birth, and that the extremities were sometimes quite blue. The abdomen was distended and hard to the touch in the regio-epigastrica, but in the left hypochondrium was to be felt a hard body, which could not be taken for any thing but the liver. On the left side of the chest percussion yielded a normal sound, whilst, by auscultation, was plainly perceptible the respiration puerilis and the beats of the heart. On the right side of the chest, on the contrary, there was, with the exception of the regio subclavicularis, no respiratory murmur, but the pulsation of the heart was very strong and perceptible on an extensive space, percussion was dull almost in the whole extent of that side, and also under (on ?) the sternum and beyond it to the left side. The writer supposed, from these facts, not only that there existed a hypertrophia cordis, but also, that the heart was situated in the right side, and he prescribed small doses of calomel and digitalis, and embrocations of iodate of mercury on the abdomen. The child, which hitherto was very restless, appeared in fact to become more quiet during the administration of these remedies, and to acquire a more lively color; the heart beat less violently, and the abdomen diminished in size. But on the night of the 17th of November the child died suddenly, as two teeth were about to make their appearance. At the post mortem examination, the heart, of the size of a man's fist, was found in the right side of the chest, the coronal veins much developed; both ventricles filled with black, fluid blood; the foramen ovale open; the great vessels, particularly the aorta, which bent to the right, much dilated; the right lung was pushed entirely behind the heart, was very small, and had two lobes; the left lung covered the heart partially, and had three lobes; both lungs were spotted black, like marble, studded with whitish-yellow smooth points, of the size of peas, and contained much blood. Nearly one-half of the cavity of the abdomen was filled with the liver; in the centre of the abdominal cavity, below the processus xyphoideus was the incisura interlobuluris, with the ligamentum teres; a little further to the right the large gall bladder; no other abnormities of the liver. The spleen in the right hypochondrium behind the liver and fastened to the fundus of the stomach. The portia pylorica of the stomach, with the duodenum, lay in the left side, and all the abdominal intestines were consequently removed from their natural position.

